# Amyloid-β Impairs Vesicular Secretion in Neuronal and Astrocyte Peptidergic Transmission

**DOI:** 10.3389/fnmol.2017.00202

**Published:** 2017-06-28

**Authors:** Virginia Plá, Neus Barranco, Esther Pozas, Fernando Aguado

**Affiliations:** ^1^Department of Cell Biology, Physiology and Immunology, University of BarcelonaBarcelona, Spain; ^2^Institute of Neurosciences, University of BarcelonaBarcelona, Spain

**Keywords:** Alzheimer’s disease, BDNF, cerebral cortex, dense-core vesicles, exocytosis

## Abstract

Regulated secretion of neuropeptides and neurotrophic factors critically modulates function and plasticity of synapses and circuitries. It is believed that rising amyloid-β (Aβ) concentrations, synaptic dysfunction and network disorganization underlie early phases of Alzheimer’s disease (AD). Here, we analyze the impact of soluble Aβ_1–42_ assemblies on peptidergic secretion in cortical neurons and astrocytes. We show that neurons and astrocytes differentially produce and release carboxypeptidase E (CPE) and secretogranin III (SgIII), two dense-core vesicle (DCV) markers belonging to the regulated secretory pathway. Importantly, Aβ_1–42_, but not scrambled Aβ_1–42_, dramatically impairs basal and Ca^2+^-regulated secretions of endogenously produced CPE and SgIII in cultured neurons and astrocytes. Additionally, KCl-evoked secretion of the DCV cargo brain-derived neurotrophic factor (BDNF) is lowered by Aβ_1–42_ administration, whereas glutamate release from synaptic vesicle (SVs) remains unchanged. In agreement with cell culture results, Aβ_1–42_ effects on CPE and SgIII secretion are faithfully recapitulated in acute adult brain slices. These results demonstrate that neuronal and astrocyte secretion of DCV cargos is impaired by Aβ *in vitro* and *in situ*. Furthermore, Aβ-induced dysregulated peptidergic transmission could have an important role in the pathogenesis of AD and DCV cargos are possible candidates as cerebrospinal fluid (CSF) biomarkers.

## Introduction

Alzheimer’s disease (AD) is by far the most common cause of dementia in the elderly. The characteristic clinical phenotype of AD is a gradual and progressive loss of memory and cognition (Scheltens et al., 2016). Accumulation of abnormally folded amyloid-β (Aβ) peptides in extracellular plaques and hyperphosphorylated tau proteins in intracellular tangles are two major pathological hallmarks of AD. However, neuritic plaques and neurofibrillary tangles are only weakly correlated with the degree of dementia in AD patients (Selkoe and Hardy, 2016). In contrast, decreased synapse number is the major quantitative correlate of loss of memory and cognition in AD brain (DeKosky and Scheff, 1990). Accordingly, a growing body of electrophysiological, biochemical and behavioral evidence suggests that synaptic dysfunction and network disorganization centrally underlie the progressive cognitive manifestations of the clinical AD occurring before the onset of symptoms (Mucke and Selkoe, 2012; Palop and Mucke, 2016).

It has been shown that the concentration of soluble Aβ, but not insoluble Aβ deposits, is a predictor of synaptic changes in AD and tracks the disease progression and cognitive decline (Lue et al., 1999; Koss et al., 2016). In fact, soluble Aβ species, mainly Aβ_1–42_ oligomers, exert a pivotal role in the pathogenesis of the synaptic damage at early stages of AD (Ferreira et al., 2015; Viola and Klein, 2015; De Strooper and Karran, 2016). Binding of Aβ to neuronal and glial plasma membranes causes multiple aberrant effects that could trigger synaptic failure, such as dysfunction of Ca^2+^ homeostasis, axonal transport, neurotransmitter receptors and transporters and mitochondria. Moreover, several studies have proposed that Aβ peptides can affect synaptic function by altering vesicular release of classical transmitters (i.e., glutamate) from neurons and astrocytes (Arias et al., 1995; Abramov et al., 2009; Parodi et al., 2010; Brito-Moreira et al., 2011; Talantova et al., 2013; Hascup and Hascup, 2016). In this regard, two recent studies showing that Aβ oligomers directly impair SNARE complex formation and synaptic vesicle (SV) exocytosis further support a deleterious function of aberrant Aβ on transmitter secretion (Russell et al., 2012; Yang et al., 2015).

Besides SVs, the so-called dense-core vesicle (DCVs, secretory granules in endocrine cells) store a wide array of neuropeptides, hormones and growth factors that enable peptidegic transmission. In neurons, and as recently proposed astrocytes, DCVs-containing transmitters budding from trans-Golgi network mature during transport along microtubules toward the cell surface and secrete their cargos by Ca^2+^-triggered exocytosis (Gondré-Lewis et al., 2012; Araque et al., 2014). Although structure and function of synapses and networks critically depend on the adjusted peptidergic transmission (van den Pol, 2012), secretory features of DCVs in the normal and pathological central nervous system have been little studied. Here, we determined the impact of Aβ on secretion of DCV cargos in cortical neurons and astrocytes. Therefore, we analyzed *in vitro* and *in situ* release of carboxypeptidase E (CPE) and secretogranin III (SgIII), two established DCVs markers which are aberrantly accumulated in neurons and astrocytes in the cerebral cortex of AD patients and amyloid-forming transgenic mice (Plá et al., 2013). First, we show that neurons and astrocytes produce distinctive forms of CPE and SgIII, which undergo release via differential mechanisms. Importantly, basal and regulated secretions of endogenously produced CPE and SgIII, as well as brain-derived neurotrophic factor (BDNF), are dramatically impaired by Aβ both in cultured dispersed cells and acute brain slices. The present results indicate that DCVs secretion is a significant target of amyloidogenic Aβ forms. Moreover, a participation of Aβ-induced peptidergic secretion alterations in the pathogenesis of AD and its potential use as a cerebrospinal fluid (CSF) biomarker are suggested.

## Materials and Methods

### Antibodies and Reagents

Monoclonal and polyclonal antibodies against CPE were obtained from BD Transduction Laboratories (San Jose, CA, USA) and GeneTex (Irvine, CA, USA). Polyclonal antibodies against SgIII were purchased from Sigma-Aldrich (Madrid, Spain). Aβ monoclonal antibodies, clones 4G8 and 6E10, were from Covance (Emeryville, CA, USA). Polyclonal PC1/3 and PC2 were from Thermo Fisher Scientific (Madrid, Spain) and kindly provided by Dr I. Lindberg (University of Maryland), respectively. Antibodies against GFAP, MAP-2, CD11b, Tuj1, β-actin and Iba1 were from Millipore Iberica (Madrid, Spain), Serotec (Oxford, UK), Sigma-Aldrich and Wako GmbH (Neuss, Germany). DL-*threo*-β-benzyloxyaspartic acid (TBOA) was from Tocris Bioscience (Bristol, UK). Most chemicals and cell culture reagents were obtained from Sigma-Aldrich and Gibco (Thermo Fisher Scientific), respectively.

### Animals and Ethics Statement

CD1 mice were provided by Envigo Rms (Sant Feliu de Codines, Spain), kept under controlled temperature (22 ± 2°C), humidity (40%–60%), and light (12-h cycles). All animals were handled in accordance with the guidelines for animal research set out in the European Community Directive 2010/63/EU, and all procedures were approved by the Ethics Committee for Animal Experimentation (CEEA), University of Barcelona (Barcelona, Spain). All efforts were made to minimize the number used and animal suffering.

### Primary Cell Cultures and Acute Brain Slices

Astroglial and neuronal cultures were obtained from CD1 mice and prepared as described previously (Paco et al., 2009). Astrocyte cultures were prepared from the whole cerebral cortex of P0-P1-day-old mice. Cortical tissues were isolated, meninges were carefully dissected away, minced and incubated in 0.5% trypsin and 0.01% DNase. Dissociated cells were seeded in flasks and grown in high-glucose Dulbecco’s Modified Eagle’s Medium and F-12 (1:1) containing 10% fetal bovine serum, 10 mM HEPES and penicillin/streptomycin at 37°C in a 5% CO_2_ incubator. At confluence (10–12 days), flasks were shaken overnight and the cells were rinsed, detached and subcultured at 1 × 10^5^ cells/cm^2^ onto poly-D-lysine-coated plastic culture dishes and glass coverslips. Under these conditions, cell cultures were essentially formed by astrocytes (>95% GFAP+), a small percentage of microglia (<5% CD11b+) and virtually devoid of neurons (<0.5% Tuj-1+). Neuronal cultures were grown from either whole cerebral cortex (including hippocampus) or isolated hippocampus of E16-E17 mouse embryos. After trypsin and DNase treatment, dissociated cells were seeded at 1.5 × 10^5^ cells/cm^2^ onto poly-D-lysine-coated culture plates and glass coverslips. Neurons were grown in Neurobasal A medium containing B27 and 1% FBS (Thermo Fisher Scientific), glutamine and penicillin/streptomycin at 37°C in a 5% CO_2_ atmosphere for 10 days. During the first 4 days, cultures were also supplemented with 20 μg/mL 5-Fluoro-2′-deoxyuridine and 50 μg/mL Uridine (Sigma-Aldrich) to inhibit mitotic activity of glial cells. Tuj-1 and MAP2 immunostaining showed that more than 95% of the cells were neurons, whereas a <5% were GFAP+ astrocytes.

Brain slices were obtained from anesthetized adult mice (ketamine 120 μg/g and xylazine 6 μg/g i.p.), as described previously (Aguado et al., 2002). Their brains were removed and placed in cold artificial CSF (ACSF) containing (in mM): NaCl 120, KCl 3, D-glucose 10, NaHCO_3_ 26, NaH_2_PO_4_ 2.25, CaCl_2_ 2, MgSO_4_ 1, pH 7.4, bubbled with 95% O_2_ and 5% CO_2_. Horizontal tissue slices (300 μm thick) were cut with a vibratome, stabilized and transferred to a release chamber. All the experiments were conducted in ACSF bubbled continuously with 95% O_2_ and 5% CO_2_ at room temperature (22–25°C).

### Aβ Aggregation and Cell Viability

Synthetic human Amyloid-β_1–42_ (Aβ) peptide (H-1368), and peptide comprised of the same amino acid composition of but in a randomized sequence, Scrambled Amyloid-β_1–42_ (H-7406; ScAβ), used as a control, were purchased from Bachem (Bubendorf, Switzerland) and prepared as described previously (Dahlgren et al., 2002). Lyophilized Aβ or ScAβ peptides were initially dissolved to 1 mM in 1,1,1,3,3,3-Hexafluoro-2-propanol (Sigma-Aldrich) and separated into aliquots in sterile microcentrifuge tubes. Then, hexafluoroisopropanol was evaporated under low temperature vacuum in a Speed Vac, and the peptide film was stored desiccated at −80°C until use. For the assembly, the peptide was first resuspended in anhydrous sterile dimethylsulfoxide (Sigma-Aldrich) to a concentration of 5 mM, diluted to a final concentration of 100 μM in 10 mM HCl and incubated for 24 h at 37°C. Aggregated species in Aβ stocks were identified by western blotting. Cultured cells and brain slices were treated with either 5 μM Aβ/ScAβ preparation or an equal volume of vehicle solution (controls). Cell viability was determined by WST-1 (Roche, Basel, Switzerland), lactate dehydrogenase (Roche) and propidium iodide/Hoechst (Sigma-Aldrich) assays. Levels of reduced WST-1 and released lactate dehydrogenase were measured with an ELISA plate reader (Tecan, Männedorf, Switzerland) at 450 nm and 492 nm, respectively. Propidium iodide/Hoechst uptake was analyzed by fluorescence microscopy and analyzed with ImageJ software.

### Release Assays

Secretion in cultured cells was assayed in 12-well culture plates except for BDNF, for which it was done in 100 mm dishes and for glutamate, for which 48-well plates were used. Poly-D-lysine-attached cells were serum and supplement starved prior to release experiments. Release assays in brain slices were performed in superfused or static chambers (displaying the same results). Secretion from cultured cells was performed in commercial media and, when K^+^ and Ca^2+^ concentrations were modified, in ACSF, whereas the release from brain slices was always carried out in ACSF. The composition of the 55 mM K^+^ ACSF was adjusted to maintain the osmolarity with a corresponding NaCl decrease. In cultured cells, conditioned media were collected and cells were washed in phosphate buffer saline (PBS) and homogenized in lysis buffer (see below). Cell media and superfusate and static ACSF from brain slices were centrifuged at 600 *g* for 5 min to remove dislodged cells and all samples were stored at −20°C. Proteins in all release samples were precipitated with 5% trichloroacetic acid, using sodium deoxycholate as a carrier, or concentrated by Amicon^®^ Ultra-15 and −0.5 Centrifugal filter devices (Merck Millipore, Madrid, Spain).

CPE and SgIII were detected by western blotting (see below) and Prep Cell Protein Standard was used as a control for the precipitation protocol for conditioned media (Bio-Rad Laboratories, Hercules, CA, USA). In cell culture media, β-actin was used to normalize the secretion in order to minimize variations in cell quantity. Levels of BDNF were quantified using the BDNF EMAX^®^ ImmunoAssay System according to the manufacturer’s instructions (Promega Corporation, Madison, WI, USA). Glutamate levels were measured using Amplex Red Glutamic Acid/Glutamate Oxidase Assay kit (Molecular Probes, Eugene, OR, USA) following the manufacturer’s protocol. BDNF and glutamate levels were normalized by total protein levels.

### Western Blotting

Cultured cells and tissues were homogenized in ice-cold lysis buffer containing 50 mM Tris-HCl pH 7.4, 150 mM NaCl, 5 mM MgCl_2_, 1 mM ethyleneglycol-bis(2-aminoethylether)-N,N,N′,N′-tetra acetic acid (EGTA), 1% Triton X-100, and protease inhibitor cocktail (Roche Diagnostics). Samples of conditioned media and postnuclear lysates were electrophoresed in 8%–12% sodium dodecyl sulfate-polyacrylamide gel electrophoresis (SDS-PAGE; Bio-Rad Laboratories) and then transferred to PVDF membranes (Bio-Rad Laboratories). The membranes were activated and blocked in a solution containing 5% nonfat milk powder in tris-buffered saline tween-20 (140 mM NaCl, 10 mM Tris-HCl, pH 7.4, and 0.1% Tween 20; TBS-Tween) for 1 h at room temperature and then incubated with primary antibodies in blocking buffer for 2 h at room temperature or overnight at 4°C. After several washes in TBS-Tween solution, the membranes were incubated for 1 h with horseradish peroxidase-conjugated secondary antibodies (Bio-Rad Laboratories). Bound antibodies were visualized with enhanced chemiluminescence reagents (Bio-Rad Laboratories). Blot images were scanned and densitometric analyses were performed using ImageJ software.

### Immunocytochemistry

Cells grown on glass coverslips were washed in ice-cold PBS and fixed with 4% paraformaldehyde in PB for 15 min. Animals were perfused transcardially under deep ketamine/xylacine anesthesia with 4% paraformaldehyde in 0.1 M PB, pH 7.4. The brains were removed from skulls, postfixed for 4 h in the same fixative solution, and cryoprotected overnight at 4°C by immersion in a 30% sucrose solution in 0.1 M PB. Forty-micrometer thick frozen sections were obtained with a cryostat and collected in PBS. Sections processed for the peroxidase method were soaked for 30 min in PBS containing 10% methanol and 3% H_2_O_2_ and subsequently washed in PBS. To suppress nonspecific binding, cell cultures and brain sections were incubated in 10% serum-PBS containing 0.1% Triton X-100, 0.2% glycine and 0.2% gelatin for 1 h at room temperature. Incubations with primary antibodies were carried out overnight at 4°C in PBS containing 5% fetal bovine serum, 0.1% Triton X-100 and 0.2% gelatin. Some histological sections were processed using the avidin-biotin-peroxidase method (Vectastain ABC kit, VECTOR, Burlingame, CA, USA). The peroxidase complex was visualized by incubating the sections with 0.05% diaminobenzidine and 0.01% H_2_O_2_ in PBS. Sections were mounted, dehydrated and coverslipped in Eukitt. Cell cultures and some brain sections were processed for immunofluorescence using secondary fluorochrome-conjugated antibodies (Alexa Fluor 488 and Alexa Fluor 568, Molecular Probes, Eugene, OR), and cell nuclei were stained with 4′,6-diamidino-2-phenylindole (DAPI, Molecular Probes, Eugene, OR, USA). Cell-containing coverslips and histological sections were mounted with Mowiol. The specificity of the immunostaining was tested by omitting the primary antibodies or by replacing them with an equivalent concentration of nonspecific IgG. No immunostaining was observed in these conditions. Bright field and fluorescent images were obtained with the Olympus fluorescent BX-61 and Leica TCS SPE scanning confocal microscopes.

### Quantitative Real-Time PCR

RNA from cells was isolated by treatment with Trizol^®^ reagent (Invitrogen) following the manufacturer’s instructions and the quantity and quality were determined with a NanoDrop ND-1000 (NanoDrop Technologies, Wilmington, DE, USA) and Bioanalyzer 2100 (Agilent, Waldbronn, Germany). cDNA was synthesized using the Superscript III Reverse Transcriptase kit (Invitrogen) from 1 μg of total RNA. Reactions were incubated at 25°C for 10 min, 50°C for 30 min, 85°C for 5 min, chilled on ice and finally *E. coli* RNAse H was added and incubated at 37°C for 20 min. Quantitative real-time PCR (qPCR) was performed using the StepOneTM Real-Time PCR System (Applied Biosystems) using TaqMan Probes Mm00516341_m1 (CPE), Mm00485961_m1 (SgIII) and Mm01277042_m1 (TBP, as housekeeping gene). The 20 μl PCR included 0.01 μl RT product, 1× PerfeCTa^®^ qPCR FastMix^®^ II with ROX (Quanta BioSciences, Inc.) and 1 μL TaqMan probe. The reactions were incubated in a 48-well plate at 95°C for 5 min, followed by 42 cycles of 95°C for 15 s, 58°C for 15 s and 72°C for 30 s. All reactions were run in triplicate. The threshold cycle (C_T_) is defined as the fractional cycle number at which the fluorescence passes the fixed threshold.

### Statistical Analysis

Data are shown as the mean ± Standard Error of the Mean (SEM) summarizing three or more independent experiments, performed at least in triplicates. Non-parametric one-way ANOVA were calculated to determine significant effects of treatments, using Kruskal-Wallis or Friedman test when appropriate. Changes were calculated in relation to the average of controls using Mann–Whitney or Wilcoxon tests as *post hoc* analysis. Significance was set at *p < 0.05, **p < 0.01 and ***p < 0.001.

## Results

### Differential Mechanisms Underlie CPE and SgIII Secretion from Neurons and Astrocytes

**Figure 1:**
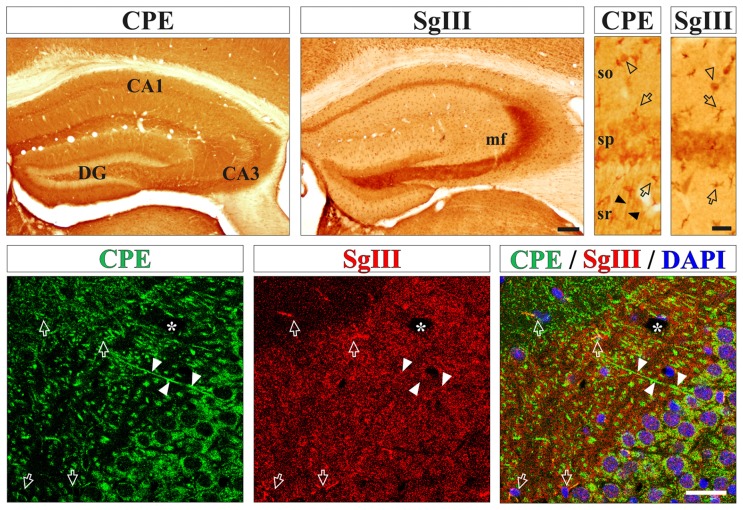
Neurons and astrocytes abundantly express carboxypeptidase E (CPE) and SgIII proteins in the cerebral cortex *in vivo*. Upper boxes illustrate immunoperoxidase staining for CPE and SgIII in panoramic (left) and CA1 region (right) views of the hippocampal formation. Both proteins display a remarkable area specific and laminar distribution. Neuronal CPE is primarily found in dendritic shafts (filled arrowheads) and perikarya, where mossy fibers robustly exhibit SgIII. Intense immunolabeling for SgIII and CPE is detected in astroglial cell bodies and processes (arrows). Bottom boxes show confocal images of CPE (green) and SgIII (red) double immunofluorescences in the hippocampal CA3 region. Both proteins are mainly associated with different neuronal domains. CPE is largely located in somatodendritic compartments and SgIII is mainly distributed in terminal-like buttons of mossy fibers. Intense SgIII and CPE immunofluorescences colocalize in the same astrocyte processes (arrows). Nuclei are stained in blue. Arrows and arrow-heads point to astroglial cells and interneurons, respectively. Filled arrow-heads outline dendrites and asterisk indicates blood vessels. Scale bars in μm: upper-left, 250; upper-right and bottom, 50. Abbreviations: CA, regions of the hippocampus; DG, dentate gyrus; mf, mossy fibers; so, stratum oriens; sp, stratum pyramidale; sr, stratum radiatum.

First, we determined the *in situ* cellular location of the DCV markers CPE and SgIII in the mouse cerebral cortex by immunohistological methods. High levels of both proteins were found in processes and cell bodies of pyramidal and non-pyramidal neurons and astrocyte-like glial cells (Figure 1). For neuronal CPE and SgIII, we detected differential location patterns at the regional and subcellular levels. In general, CPE immunostaining was more intense and more extended than for SgIII (Figure 1). Both proteins were present in perikarya, but only CPE was associated with dendritic shafts. Characteristically, SgIII was found abundantly as immunoreactive puncta throughout the neuropil, resembling axon terminals (Figure 1). Differential location of these two DCV proteins in neurons was apparent for the CA3 region of the hippocampus, where CPE- and SgIII-labeled projections corresponded with dendrites and mossy fibers, respectively (Figure 1). Regarding glial cells, most cortical astrocyte-like somata through the gray and white matters copiously displayed both CPE and SgIII (Figure 1). Double immunofluorescence showed that non-neuronal CPE and SgIII were associated with virtually all GFAP+ astrocytes and absent in Iba1+ microglial cells (data not shown), in agreement with our previous report (Paco et al., 2010).

**Figure 2:**
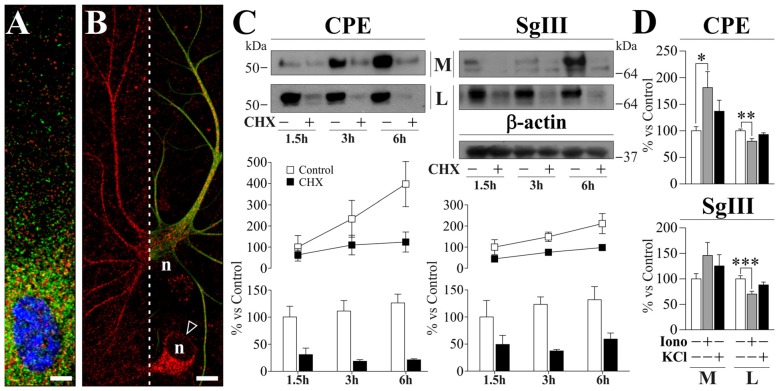
Localization and release of CPE and SgIII in cultured cortical astrocytes. **(A,B)**. Confocal immunofluorecence images in astrocytes grown in glial **(A)** and neuronal **(B)** enriched cultures. High magnification in **(A)** shows a poor co-localization (yellow signal) of CPE (green) and SgIII (red) around the nucleus (blue). The astrocyte identity of a process-bearing cell positive for SgIII (red) is revealed by GFAP (green) co-staining (right in **B**). In **(B)**, arrow-head points to a neuronal soma displaying SgIII (red) and *n* indicates nucleus location. Scale bars in μm: **(A)** 5; **(B)** 15. **(C)** Immunoblots illustrate cellular content (lysates, L) and basal secretion into the medium (M) of CPE and SgIII in astrocyte cultures treated or not with 7.5 μM CHX during 1.5 h, 3 h and 6 h. β-actin was used as loading control. The graphs below summarize percent variation of the extracellular (lines) and intracellular (bars) levels of CPE and SgIII after CHX administration compared to controls. Data are presented as the mean ± standard error of the mean (SEM) of a representative experiment performed in quadruplicates. **(D)** Histograms summarizing the effect of 1 μM ionomycin (Iono) and 55 mM KCl incubations over 15 min on release (M) and cell content (L) of CPE and SgIII. Values represent percent variation compared with controls and are presented as the mean ± SEM. All the data (M and L) was normalized by intracellular levels of β-actin. *p < 0.05; **p < 0.01; ***p < 0.001, Mann-Whitney test.

To study peptidergic secretion from astrocytes and neurons, we prepared cortical primary cultures highly enriched in each cell type. Astrocyte cultures were virtually devoid of neurons, while a small number of astrocytes (<5%) was present in neuronal cultures which improved survival. In cultured astrocytes, CPE and SgIII were associated with secretory organelles showing a non-overlapping location, mainly for distal vesicles (Figure 2A). CPE- and SgIII-immunolabeled vesicular compartments were also evident in astrocytes grown within neuronal cultures (Figure 2B). GFAP co-labeling was used to validate astroglial identity. Careful analysis of media and cell lysates of glial cultures by western blotting revealed that astrocyte CPE and SgIII proteins corresponded to the nonprocessed forms (~55 kDa for CPE and ~80–75 kDa for precursor SgIII, pSgIII; Figure 2C). Because glial-produced proteins likely corresponded to the uncleaved precursor forms, we determined whether astrocytes lacked the corresponding PC1/3 and PC2 processing prohormone convertases. Double immunocytochemical labeling and Western blotting showed that *in vitro* astrocytes did not express either PC1/3 or PC2 proteins (data not shown).

As previously reported for SgIII (Paco et al., 2010), we show here that cultured astrocytes displayed high rates of basal release of both CPE and SgIII (Figure 2C). To analyze secretory kinetics of *de novo* synthesized CPE and SgIII in astrocytes, extracellular and intracellular protein pools were analyzed during cycloheximide (CHX) chase. As expected for secretory proteins, untreated cells showed rising extracellular levels and steady intracellular pools of CPE and SgIII over time. When protein synthesis was blocked by CHX, decreasing levels of intracellular CPE and SgIII were coupled with an almost invariable secreted pool (Figure 2C). These observations show that newly generated DCVs-like in astrocytes are poorly retained and rapidly undergo exocytosis, independently of stimuli. Next, we evaluated the regulated secretion of glial CPE and SgIII triggering [Ca^2+^]_i_ elevation by ionophores. Noteworthy, exposure to 1 μM ionomycin over 15 min gave variable responses from one culture set to another. Compared to unstimulated cultures, released CPE was higher after ionophore administration (181.4 ± 29.8% over basal; *p* = 0.03), while no statistically significant changes were observed for SgIII during stimulation (146.1 ± 25.4% over basal; *p* = 0.3). Moreover, CPE and SgIII intracellular levels were decreased after treatment (Figure 2D). Finally, the addition of 55 mM KCl to the media did not substantially change the levels of CPE nor SgIII secreted from astrocytes (Figure 2D). We conclude that cultured astrocytes robustly produce unprocessed forms of CPE and SgIII and largely release them in a stimulus-independent fashion.

**Figure 3:**
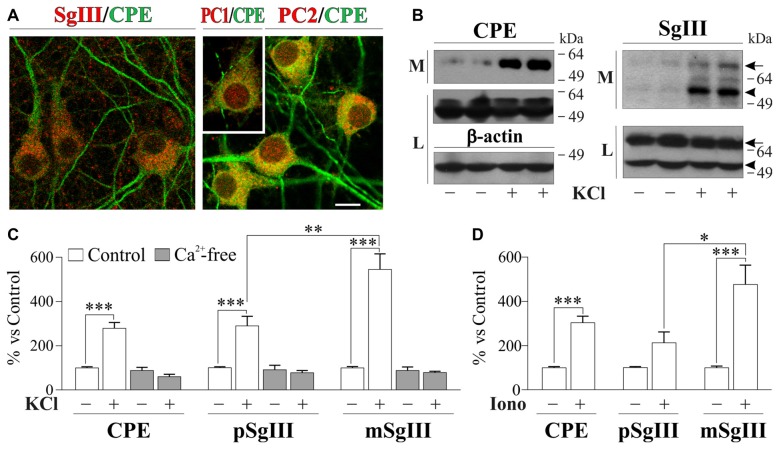
Cellular distribution and release of CPE and SgIII in cultured neurons of the cerebral cortex. **(A)** Confocal immunofluorescence shows a prominent dendritic location for CPE (green) in pyramidal- and stellate-shaped neurons, while SgIII (red, left) is found in cell bodies and axon-like processes. No apparent colocalization was observed for the 2 proteins. Abundant signal shows the presence of the PC1/3 (red, inset) and PC2 (red, right) in the cultured neurons, with a broad somatic distribution. Scale bar in μm: 10. **(B)** Western blots show a very low basal secretion for CPE and SgIII, which were abundantly released in response to depolarization by KCl 55 mM for 15 min. Both forms of SgIII (arrow: proform, p and arrowhead: mature, m) were found in the extracellular media (M). No changes were found at intracellular levels in the lysate fraction (L). β-actin was used as loading control. **(C)** Release in response to KCl-depolarization was significant for CPE and both *p*- and mSgIII, the latter being especially high. In all cases, evoked secretion was abolished in absence of Ca^2+^, showing a Ca^2+^ dependance. **(D)** 1 μM Ionomycin was used to trigger the CPE and SgIII release. Graphs show the percent variation compared with controls and are presented as the mean ± SEM normalized by intracellular levels of β-actin. *p < 0.05; **p < 0.01; ***p < 0.001, Mann-Whitney test.

In neuronal cultures, CPE and SgIII were associated with secretory organelles of pyramidal- and stellate-shaped neurons (Figure 3A). In agreement with the above *in vivo* data, a preferential location in dendrites was observed for CPE, whereas SgIII was mainly associated with axon-like projections and terminals. Neuronal and dendritic identities were confirmed by double immunolabeling with MAP2. In contrast to glial cells, cultured neurons produce and release both the precursor and mature forms of CPE (~55 and 53 kDa) and SgIII (~75 and 55 kDa; pSgIII and mSgIII respectively; Figure 3B). Moreover, as anticipated by the mature form occurrence of CPE and SgIII, cultured neurons abundantly displayed the DCV-associated convertases PC1/3 and PC2 (Figure 3A).

Opposite to astrocytes, cultured neurons showed a very low basal and high stimulus-triggered secretion of CPE and SgIII (Figures 3B–D). Therefore, forcing Ca^2+^ entry during 15 min by 1 μM ionomycin addition increased up to fivefold secretion from neuronal cells (Figure 3D). Depolarization by KCl for 15 min resulted in a dramatic enhancement of CPE and SgIII release (Figure 3C). Although shorter stimulation times, such as 5 min, offered similar results, 15 min of depolarization was maintained to ensure detection of released proteins by Western blotting. K^+^-evoked secretion of CPE was enhanced by a 279% over basal, whereas a 289% was observed for SgIII forms. Because nominally Ca^2+^-free medium virtually abolished DCVs release, K^+^-induced release of CPE and SgIII in cultured neurons entirely depended on the influx of this cation (Figure 3C). Interestingly, Ca^2+^-evoked SgIII secretion was stronger for mature forms than for precursors in both ionomycin (1.7 m/p ratio) and KCl (2.0 m/p ratio) stimulations. These data indicate that neuronal and astroglial DCVs undergo differential proteolytic processing and exocytotic profiles.

### Aβ Alters Production and Release of CPE and SgIII in Cultured Astrocytes

**Figure 4:**
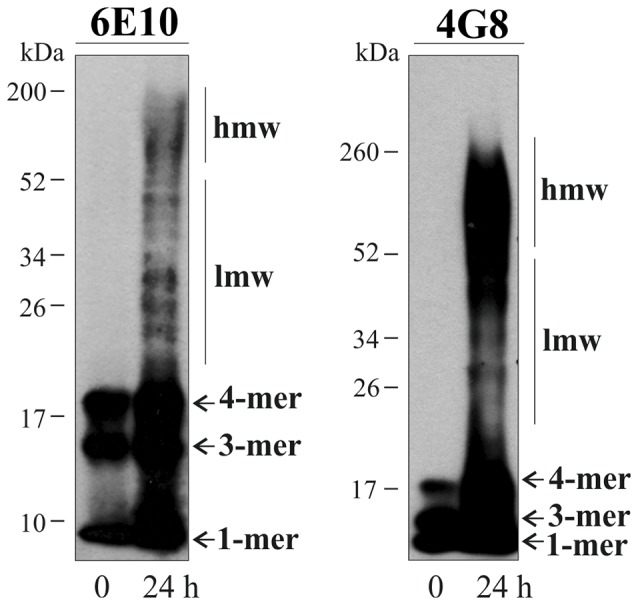
Characterization of aggregates present in the Aβ preparations. Immunoblots illustrate 6E10- and 4G8-immunoreactive soluble Aβ_1–42_ peptide before and after 24 h incubation at 37°C. Monomeric (1-mer), trimeric (3-mer) and tetrameric (4-mer) forms of Aβ are detected in both preparations, whilst low molecular weight (lmw) and high molecular weight (hmw) aggregates are only detected after the 24 h-incubation.

To determine whether Aβ alters glial and neuronal DCVs secretion, we prepared Aβ assemblies incubating Aβ_1–42_ peptides for 24 h at 37°C. Immunoblotting with 6E10 and 4G8 Aβ antibodies revealed that Aβ preparations contained a broad mixture of low- (20–50 kDa) and high-molecular-weight (>50 kDa) aggregates, as well as the Aβ monomers, trimers and tetramers (Figure 4). Consistent with previous reports (Moreth et al., 2013), mono-tetrameric species were instantaneously formed, whereas larger oligomeric aggregates appeared over time of aging. No immunoreactive bands were detected from ~200 kDa to the top/entrance of the gels (Figure 4).

Cell viability of cultured cells incubated with 5 μM Aβ was evaluated at 24 h for astrocytes and 16 h for neurons. Cellular membrane integrity was analyzed by a propidium iodide/Hoechst uptake test. No changes were observed between Aβ-treated and untreated cell cultures (Aβ vs. control: 99.6 ± 0.7%, *p* = 0.8 for neurons and 100.2 ± 0.8%, *p* = 0.9 for astrocytes). Due to the vulnerability of neurons, 2 additional tests were performed. WST-1 reduction was evaluated to detect variations in the mitochondrial metabolic rate, finding no significant changes (111.7 ± 4.7% of the control, *p* = 0.14). Additionally, the release of the cytoplasmic enzyme lactate dehydrogenase was analyzed in neuronal supernatant, showing no increase in response to Aβ (97.7 ± 3.6% of the control, *p* = 0.6). Because no differences were found between Aβ- and vehicle-treated cultures in any of the tests performed, no toxicity was found at the incubation times used.

**Figure 5:**
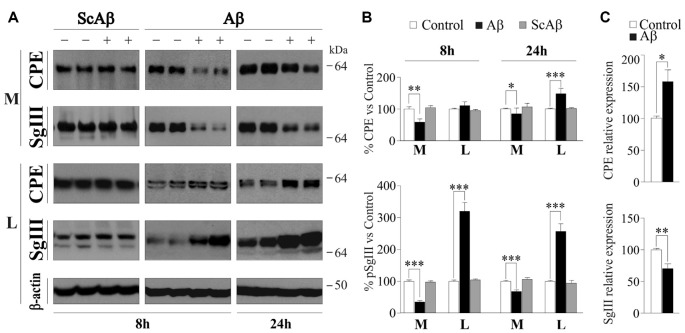
Basal release and production of CPE and SgIII is altered in Aβ-treated cultured astrocytes. **(A)** Western blots showed basal release from cortical astrocytes treated with 5 μM Aβ during 8 or 24 h. A marked reduction in the extracellular levels (media, M) of SgIII and CPE compared with controls and an intracellular (lysates, L) accumulation is found in response to treatment with Aβ. No changes were found in response to ScAβ, either in the extracellular medium or the content. β-actin was used as loading control. **(B)** Graphical representation of the levels of CPE and SgIII compared with the vehicle-treated levels. Data are presented as the β-actin-normalized mean ± SEM. **(C)** mRNA quantification by Quantitative Real-Time PCR (qPCR) of CPE and SgIII of Aβ-treated cells (16 h) compared with the vehicle-treated levels. Values represent percent variation compared with controls and are presented as the mean ± SEM. *p < 0.05; **p < 0.01; ***p < 0.001, Mann-Whitney test.

Next, we analyzed the effect of Aβ on astrocyte CPE and SgIII secretion. Because the weak regulated release of these proteins in glia, we focused on their basal secretion at 8 and 24 h. Incubation of astrocytes with 5 μM Aβ caused a significant reduction in the extracellular levels of SgIII and CPE, mainly at 8 h (65% for SgIII and 41% for CPE) compared with controls (vehicle-treated cells; Figures 5A,B). The unchanged CPE and SgIII levels in culture media of astrocytes incubated with a scrambled amino acid sequence of Aβ_1–42_ (5 μM ScAβ) substantiated the specific effect of the aberrant amyloid on released glial proteins. To assess whether decreased levels into the media correlated with a diminished production or an impaired release, intracellular SgIII and CPE levels were assayed in Aβ- and ScAβ-treated cells. Concomitantly with a reduction in secreted CPE and SgIII, Aβ markedly increased their cellular content, mainly for SgIII (320% at 8 h and 257% at 24 h, *p* < 0.0001). No differences were detected after incubation with ScAβ peptides (Figures 5A,B). In addition to an impaired secretion, an Aβ-induced transcriptional dysregulation could contribute to change extra- and intracellular levels of secretory proteins. Therefore, we performed qPCR analysis for CPE and SgIII mRNA in Aβ-treated and control astrocytes. We found that levels of CPE transcripts were upregulated by amyloid species (158.1 ± 18.7% of control, *p* = 0.03), whereas SgIII mRNA expression was declined (70.2 ± 7.8% of control, *p* = 0.004; Figure 5C). Taking together, these results indicate that Aβ differentially regulates CPE and SgIII transcription and consistently impairs their protein secretion in astrocytes.

### Regulated Secretion of DCV Cargos from Cultured Neurons is Impaired by Aβ

**Figure 6:**
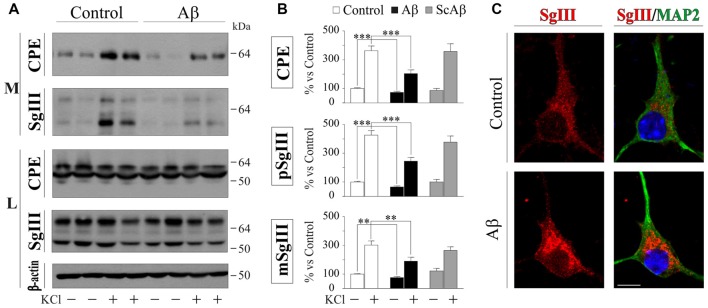
Aβ impairs regulated secretion of CPE and SgIII in cultured cortical neurons. **(A)** Western Blot analysis shows a decrease in the release of CPE and SgIII both in basal and stimulated conditions in response to β-amyloid compared with vehicle-treated controls. Intracellular levels were unaffected by the treatment. β-actin was used as loading control. **(B)** Summary of Aβ and ScAβ effects on basal and evoked secretion of CPE, pSgIII and mSgIII. Data are presented as the β-actin-normalized mean ± SEM. **p < 0.01; ***p < 0.001, Mann-Whitney test. **(C)** Confocal immunofluorescent images show an aberrant intracellular accumulation of SgIII in Aβ-treated neurons, mainly localized around the nucleus. Scale bar in μm: 10.

To evaluate the impact of Aβ on neuronal DCV release, primary cultures were exposed to vehicle (control) and 5 μM Aβ and ScAβ preparations for 16 h, then basal and K^+^-evoked release were analyzed by immunoblotting. A representative experiment in Figure 6A illustrates no differences in the intracellular levels of CPE, SgIII forms and β-actin after Aβ-treatments (data quantification not shown). However, a significant decrease of basal secretion was detected for CPE (73% of control, *p* < 0.0001) and precursor (75% of control, *p* = 0.007) and mature (66% of control, *p* = 0.0005) SgIII forms in Aβ-exposed neurons, but not in cells incubated with ScAβ (Figures 6A,B). Importantly, Aβ specifically impaired K^+^-depolarized release of CPE (Aβ 203.3% vs. control 363.2%, *p* < 0.0001), pSgIII (Aβ 190.0% vs. control 302.1%, *p* = 0.006) and mSgIII (Aβ 245.3% vs. control 426.0%, *p* < 0.0001) but not ScAβ (Figures 5A,B). Furthermore, an immunocytochemical analysis was performed on MAP2-identified neurons to examine subcellular distribution. Comparing neurons exposed to amyloid with vehicle (control), aberrant immunoreactive accumulations around the nuclei were detected in Aβ-treated cultures. This abnormal distribution was mainly associated with pyramidal-shaped cells and principally occurred for SgIII (Figure 6C). The present observations evidence that Aβ strongly alters the regulated secretory pathway in neurons, impairing the evoked release of CPE and SgIII.

**Figure 7:**
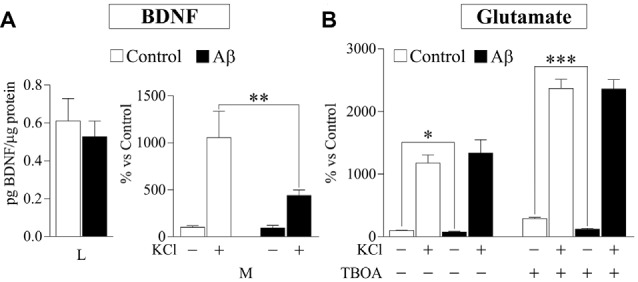
Aβ effects on brain-derived neurotrophic factor (BDNF) and glutamate release in hippocampal cultured neurons. **(A)** Intracellular BDNF content (lysates, L) in neuronal cultures was no affected by 5 μM Aβ during 16 h, whereas K^+^-stimulated release was decreased (media, M). **(B)** Extracellular glutamate levels were decreased in basal media of neurons treated with 5 μM Aβ during 16 h, whereas no changes were observed after KCl stimulation. Inhibiting glutamate re-uptake by TBOA shows that released neurotransmitter was unchanged during depolarization but decreased at basal conditions. Values were normalized by total proteins levels and represent percent variation compared with controls and are presented as the mean ± SEM. *p < 0.05; **p < 0.01; ***p < 0.001, Wilcoxon test.

Probably, the most studied DCV protein in brain-related diseases is the pleiotrophic growth factor BDNF (Adachi et al., 2014). With the aim to analyze whether release of physiologically relevant DCV cargos is affected by Aβ, we investigated secretion of endogenously produced BDNF by a sensitive sandwich immunoassay. First, we assessed BDNF levels in the same cortical-derived neuronal cultures used for CPE and SgIII analysis. However, cellular content of BDNF in cultured whole cortices was very low (8.6 ± 2.7 pg per 5×10^6^ cells). Therefore, although K^+^-evoked secretion could be determined, basal secretion was under detectable levels. In order to achieve detectable basal levels, we prepared BDNF-enriched cultures by isolating hippocampal neurons (Chen et al., 2006). Intracellular BDNF in hippocampal neurons was around four-fold higher than in whole cortical cultures (38.8 ± 4.9 pg per 5×10^6^ cells). No significant differences were found in intracellular BDNF levels in untreated and 5 μM Aβ treated cells for 16 h (control 0.61 vs. Aβ 0.53 pg BDNF/μg protein, *p* = 0.6). As shown in Figure 7, depolarization-stimulated secretion of BDNF in hippocampal neurons was greatly impaired by Aβ exposure (Aβ 439.6% vs. control 1055.0%, *p* = 0.003), whereas basal release levels were unchanged (94.8% of control, *p* = 0.5). Furthermore, immunoblot examination of CPE and SgIII secretion patterns in Aβ-treated hippocampal neurons offered similar results to those obtained in whole cortical cultures. Decrease in basal secretion was 44% for CPE (*p* = 0.0002), 23% for pSgIII (*p* = 0.003) and 53% for mSgIII (*p* = 0.01) in Aβ-treated hippocampal cultures, whereas K^+^-depolarized release was impaired by a 64.7% (*p* = 0.007), 50.9% (*p* = 0.01) and 43.4% (*p* = 0.03) for CPE, pSgIII and mSgIII, respectively.

Finally, to compare Aβ effects on neuronal DCV secretion with those on SV exocytosis, we determined glutamate release in hippocampal neurons exposed to 5 μM Aβ for 16 h by a fluorometric assay. Compared to unstimulated conditions, glutamate levels in the media were robustly increased during K^+^-evoked depolarization (1176% over basal). Incubation of Aβ caused a significant decrease in extracellular glutamate levels at basal conditions (78.1% of control), whereas no changes were observed during stimulation (Figure 7). To ascertain the reliable contribution of secretion in extracellular glutamate levels, its re-uptake was blocked by addition of the excitatory amino acid transporter inhibitor TBOA (75 μM). In all TBOA-treated samples, extracellular glutamate concentrations were higher than in non-blocked conditions. Similar to the results obtained without the transporter inhibitor, in absence of glutamate re-uptake, Aβ reduces levels of glutamate released in unstimulated cells (41.4% of control) but does not influence secretion in KCl-depolarized neurons (Figure 7). Taken together, these results show that Aβ specifically impairs Ca^2+^-regulated secretion of DCVs in neuronal populations.

### Aβ Impairs DCV Secretion in Adult Neural Cells *In Situ*

**Figure 8:**
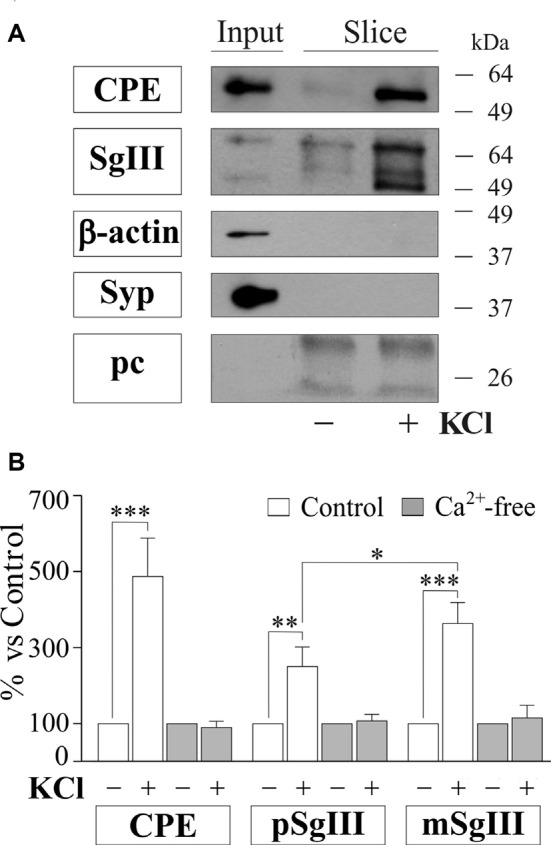
Basal and depolarization-evoked release of CPE and SgIII from adult acute brain slices. **(A)** Representative immunoblots detecting different proteins in the perfused artificial CSF (ACSF) of the same slice after 15 min of basal (−) and 55 mM KCl (+) conditions. β-actin and synaptophysin (Syp) were used to confirm slice integrity and prestained proteins were added to ensure correct sample concentration (precipitation control, pc). Forebrain homogenates were used as input. **(B)** Histograms summarize variations of released CPE, pSgIII and mSgIII after KCl stimulation in normal (2mM [Ca^2+^]_0_) and nominally Ca^2+^-free (0 mM [Ca^2+^]_0_, 2 mM EGTA) ACSF compared with basal conditions, showing mean ± SEM. *p < 0.05; **p < 0.01; ***p < 0.001, Mann-Whitney test.

To gain further insight into the impact of Aβ on regulated secretory pathway in neural cells, we next performed experiments on acute brain slices from adult mice. A major advantage of slice preparations is that cells *in situ* largely retain the states of differentiation, cytoarchitecture, extracellular matrix and synaptic circuits of the intact adult brain. First, we characterized CPE and SgIII secretion in horizontal adult brain slices under different conditions by western blot analysis (Figure 8). Low levels of both proteins were detected in unstimulated slices. However, CPE and SgIII release markedly increased after 10 min of a depolarizing stimulus (55 mM [K^+^]_0_). Cell integrity in the slice was confirmed by the lack of vesicular integral and cytosolic proteins, such as synaptophysin and actin, in the extracellular media. As occurred in cultured neurons (Figure 3C), KCl-evoked secretion of mSgIII form was stronger than for precursors (Figure 8B). To determine the involvement of Ca^2+^ in the evoked secretion of CPE and SgIII, we performed similar experiments in a nominally Ca^2+^-free ACSF. Lack of extracellular Ca^2+^ totally prevented the K^+^-induced CPE and SgIII secretion (Figure 8B). These results show that *in situ* adult neural cells of the brain release CPE and SgIII in a depolarization- and Ca^2+^-dependent manner.

**Figure 9:**
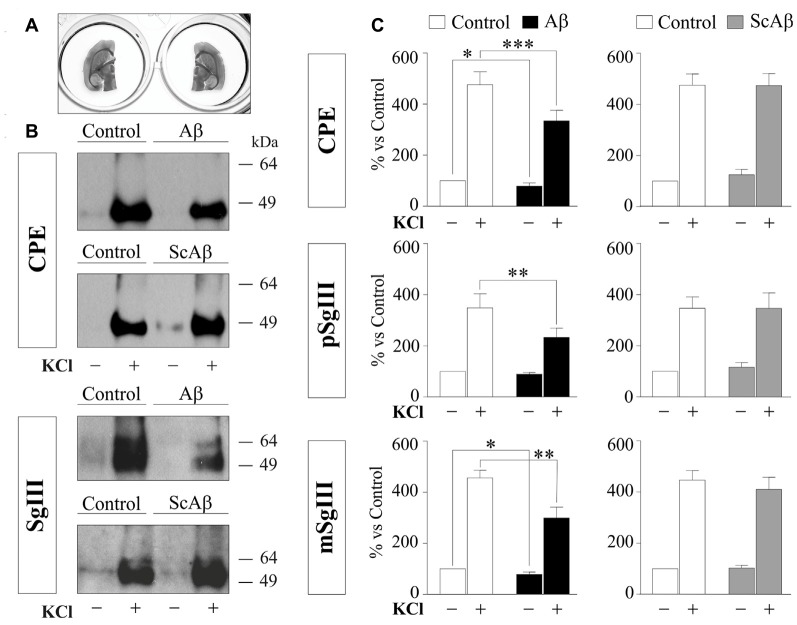
Aβ impairs CPE and SgIII secretion in acute adult brain slices. **(A)** Left and right hemispheres of the same slice were incubated in parallel with vehicle and Aβ or vehicle and ScAβ for 8 h. **(B)** Immunoblots showing released CPE and SgIII from representative paired hemispheres after 15 min of basal (−) and 55 mM KCl (+) conditions. **(C)** Summary of Aβ and ScAβ effects on basal and evoked secretion of CPE, pSgIII and mSgIII. Values represent percent variation compared with basal release of controls (mean ± SEM). Statistical significance: *p < 0.05; **p < 0.01; ***p < 0.001, Wilcoxon test.

To compare DCV secretion in the same cell populations between control and Aβ-treated brain slices, we split horizontal brain slices into left and right hemispheres (Figure 9A), minimizing the variability associated with cellular composition and responsiveness inherent to each slice. Each pair of hemispheres was incubated with vehicle (control) and 5 μM Aβ or 5 μM ScAβ for 8 h and basal and K^+^-evoked secretion of CPE and SgIII were analyzed by immunoblotting and statistical analysis was performed using a Wilcoxon test (Figures 9B,C). Aβ notably reduced depolarization-evoked release of CPE (Aβ 334% vs. control 475.9%, *p* = 0.0005), pSgIII (Aβ 232.9% vs. control 348.3%, *p* = 0.0068) and mSgIII (Aβ 299.1% vs. control 456.2%, *p* = 0.0015). Moreover, basal secretion of CPE and mSgIII was also impaired by Aβ (88.0% and 89.1% of controls, respectively). No changes were observed in hemispheric slices incubated with ScAβ (Figures 9B,C).

In summary, these results evidence that Aβ impairs DCV secretion in cultured cortical cells and adult neural networks *in situ*.

## Discussion

The major finding of this study is that aberrant Aβ markedly impairs neuronal and astrocyte secretion of endogenously-produced DCV cargos *in vitro* and *in situ*. CPE and SgIII are two established DCV markers that belong to the regulated secretory pathway of neurons and endocrine cells with recognized roles in sorting, trafficking and processing of peptidic cargos and proposed new functions as intercellular transmitters (Bartolomucci et al., 2011; Cawley et al., 2012; Cheng et al., 2014). Here we show that neurons and astrocytes produce specific CPE and SgIII forms which are released in a cell type specific manner. CPE, SgIII and BDNF secretion, but not glutamate release, is dramatically impaired by Aβ in dispersed neurons and astrocytes in culture. Furthermore, similar detrimental effects of Aβ assemblies on basal and evoked release of DCV cargos are observed on treated acute brain slices.

### Secretion of DCV Cargos in Neurons and Astrocytes

As well as their known expression by neurons, CPE and SgIII are also abundantly produced by astrocytes *in vitro* and *in vivo* (Paco et al., 2010). In agreement with a previous study performed in human brains (Plá et al., 2013), we found a segregate location of CPE and SgIII in DCV subsets in mouse neurons and astrocytes. Irrespective whether neurons were analyzed in cultures o *in situ*, a preferential somatodendritic location was observed for CPE, whereas SgIII was mainly associated with axons and terminal-like buttons. A non-overlapping vesicular location of these proteins was also found in cultured astrocytes. These observations lend support to the idea of differential routing and release of DCV cargos in the same secretory cell (Fisher et al., 1988; Zhang et al., 2011). Furthermore, the separate vesicular distribution of CPE and SgIII noticed here may imply differences in sorting mechanisms of neural cells compared with those described in endocrine cells (Hosaka et al., 2005; Cawley et al., 2016).

Although both CPE and SgIII are indeed expressed by neurons and astrocytes, we found important differences in the forms produced and their release dynamics comparing cultures of each cell type. First, probably due to the lack of the prototypical prohormone convertases of the regulated secretory pathway (PC1/3 and PC2; Winsky-Sommerer et al., 2000), astrocyte CPE and SgIII forms correspond to nonprocessed precursors. Additionally, a differential secretory profile was observed between neurons and astrocytes. Stimuli that evoked robust CPE and SgIII release in neurons barely provoked a response in astrocytes. In good agreement with a seminal work analyzing secreted CPE enzymatic activity from Fricker’s lab (Vilijn et al., 1989), we observed no response of released CPE and SgIII to elevated [KCl]_o_ from astrocytes. In addition, increasing [Ca^2+^]_i_ by ionophores caused variable and weak release responses in glial cells. Furthermore, we show that newly synthesized CPE and SgIII in non-stimulated astrocytes are poorly retained and rapidly released. Taken together, these observations would suggest that although bonafide CPE and SgIII are produced in astrocytes, they are not sorted and stored in typical DCVs. Based essentially on cell cultures, recent studies have proposed the occurrence of DCVs in astrocytes (Verkhratsky et al., 2016). However, several typical hallmarks of neuronal and endocrine DCVs (e.g., size, core density, long residence in cytoplasm, presence of synaptobrevin2, robust stimulus-dependent exocytosis) have hardly been demonstrated in cultured astrocytes (Crippa et al., 2006; Potokar et al., 2008; Paco et al., 2009). Because astrocytes *in vitro* display a partially immature phenotype and they do not accurately reproduce their *in vivo* attributes, DCV features in astroglial cells may be higher *in situ* than in culture. In fact, regulated gliosecretion of DCV components in cultured cells is enhanced under differentiating conditions, such as activation of the cAMP pathway and tone attenuation of the REST/NRSF transcription factor (Paco et al., 2009, 2016; Prada et al., 2011). On this basis, the typical size and dense core characteristics of neuroendocrine DCVs have been evidenced in granin-containing vesicles of human astrocytes *in vivo* (Hur et al., 2010).

### Peptidergic Secretion as a New Target for Aβ

Aβ dramatically impairs neuronal and astrocyte secretion of DCV cargos *in vitro* and *in situ*. In unstimulated astrocyte cultures Aβ exposure dramatically reduced levels of CPE and SgIII released over 8–24 h. Conversely, intracellular amounts were increased without an apparent correlation with transcriptional mechanisms. Due to the poor cytoplasm retention observed for exocytic vesicles, CPE and SgIII secretion decrease and intracellular accumulation induced by Aβ in astrocytes probably reflects an impairment of the secretory pathway. In neuronal cultures, overnight incubation with Aβ did not provoke apparent changes in intracellular levels of the DCV cargos CPE, SgIII and BDNF, but did affect their basal and KCl-stimulated secretion. Interestingly, basal release of the SV transmitter glutamate was also impaired by Aβ, while its evoked discharge was largely preserved. Because Aβ incubations reduce spontaneous neuronal activity of recurrent networks in primary cultures (Rönicke et al., 2011; Lee et al., 2013; Zurita et al., 2013), it is possible that the intrinsic activity-driven exocytosis of both SVs and DCVs decreases as Aβ lowers activation rates. In contrast to basal secretion, when release was forced by K^+^-induced depolarization, Aβ selectively impairs secretion of cargos from DCVs but not from SVs. Although the cell type source of secreted cargos cannot be addressed in intact neuro-glial circuitries, alterations in CPE and SgIII release in treated acute slices from adult brains further substantiate the notion that Aβ impairs peptidergic secretion in cortical cells. The present conclusion is supported by previous studies showing secretion failures in exogenous (ANP.emd) and endogenous (cystatin C and thrombospondin 1) vesicular cargos in cultured astrocytes and neurons expressing presenilins carrying mutations linked to familial Alzheimer disease and incubated with Aβ (Ghidoni et al., 2007; Rama Rao et al., 2013; Stenovec et al., 2016).

Because Aβ can induce dysfunctions in different factors and stages involved in the secretory pathway, how amyloidogenic peptides affect neural vesicular secretion is uncertain. Aβ could alter peptidergic secretion influencing vesicular biogenesis, trafficking and exocytosis. It has been shown that soluble Aβ forms induce key changes which could compromise the integrity of the secretory pathway at early stages, such as endoplasmic reticulum stress, Golgi fragmentation and autophagy (Alberdi et al., 2013; Joshi et al., 2014; Son et al., 2015). Moreover, Aβ can also impair transport, docking and discharge of secretory vesicles. Recent evidence has shown that Aβ disrupts regulated exocytosis through its direct interaction with SNARE proteins (Russell et al., 2012; Yang et al., 2015). However, given that DCVs and SVs share the basic SNARE machinery for Ca^2+^-evoked secretion (Gondré-Lewis et al., 2012), the Aβ-induced impairment in DCV cargo release from KCl-depolarized neurons and not for glutamate makes a major contribution of SNAREs unlikely. On the contrary, a failure of vesicular trafficking could underlie the secretion changes reported here. A large body of evidence indicates that defects in microtubule-mediated transport contribute to the initiation or progression of neurodegenerative diseases, including AD (Encalada and Goldstein, 2014; Llorens-Martín et al., 2014). Specifically, soluble Aβ species impair dendritic and axonal BDNF transport in cultured neurons (Decker et al., 2010; Gan and Silverman, 2015). Moreover, spontaneous and Ca^2+^-dependent mobility of ANP.emd-containing vesicles was diminished in astrocytes expressing mutated presenilin 1 (Stenovec et al., 2016). Although a dysregulation of Ca^2+^ homeostasis and mitochondrial function could also participate in secretion failure (Ferreira et al., 2015; Viola and Klein, 2015; De Strooper and Karran, 2016), we propose that impaired trafficking exerts a central role in the Aβ-mediated secretion alterations showed in this study. Furthermore, the aberrant accumulation of granin family members and CPE detected in dystrophic neurites and neuronal and astrocyte somata in the cerebral cortex of AD patients and amyloid-forming transgenic mice strongly supports an Aβ-induced impairment of vesicular transport and secretion in the peptidergic transmission (Willis et al., 2011; Plá et al., 2013). Lastly, which Aβ species are affecting neural peptidergic secretion is an intricate issue. In our Aβ preparation we virtually detected aggregates under 200 kDa. However, the array of different forms and the complex equilibrium among them at physiological conditions over time make difficult to ascertain the specific identity of the Aβ assemblies involved in peptidergic secretion failure (Jan et al., 2011; Moreth et al., 2013; Yang et al., 2017).

### Pathophysiologic Implication of Impaired Peptidergic Transmission in AD

Due to critical functions of CPE, SgIII and BDNF together with their wide distribution in the cerebral cortex, it is expected that the Aβ-induced release impairment showed here is involved in the AD pathophysiology. It has been shown that CPE and SgIII sort granins, proneuropeptides, prohormones and pro-BDNF to DCVs (Cool et al., 1997; Hosaka et al., 2005; Lou et al., 2005). Therefore, it is likely that proteins belonging to the regulated secretory pathway, at least those interacting with CPE and SgIII, are aberrantly co-secreted in the presence of Aβ. In addition, a dysregulated secretion could disturb the new extracellular functions attributed to CPE (alternatively named neurotrophic factor-alpha 1, NF-α1; Cheng et al., 2014). Moreover, the association of an uncovered CPE/NF-α1 gene mutation with AD comorbidity further connects CPE with this neurodegenerative disease (Cheng et al., 2016). Because BDNF has powerful and recognized effects on synaptic transmission, plasticity and neuronal survival and is strongly linked with AD, independently of transcriptional defects, the impact of an impaired release on neural network functions is anticipated (Adachi et al., 2014).

Beyond secretion failures for CPE, SgIII and BDNF and based on a common vesicular trafficking impairment, we suggest a more general effect of Aβ on both neuronal and astroglial peptidergic secretion. Because a rise in of soluble Aβ concentrations in early phases of AD is linked with synaptic dysfunction and network disorganization, it is conceivable that alteration of peptidergic transmission, which controls circuitry function and homeostasis, is involved in AD progression. It is worth noting that improving levels of DCV cargos (i.e., BDNF and somatostatin) partially recover AD-altered networks, preventing cognitive deficits and favoring Aβ clearance (Saito et al., 2005; Nagahara et al., 2009; Zhang et al., 2015). Moreover, the aberrant secretion and intracellular accumulation of CPE and SgIII observed in Aβ-treated and AD mouse and human brains (Plá et al., 2013) are in line with the low levels found in the CSF of AD patients by quantitative proteomics (Fagan and Perrin, 2012). Taking into account the importance of CSF biomarkers for clinical practice and trial design (Lleó et al., 2015), CSF changes based on peptidergic secretion failures could reflect synaptic dysfunction and serve as complementary diagnostic biomarkers of AD at early stages.

In summary, this study demonstrates that neuronal and astrocyte secretion of endogenous DCV proteins is impaired by Aβ *in vitro* and *in situ*. Additionally, Aβ-induced dysregulated peptidergic transmission could play an important role in the pathogenesis of AD and DCV cargos are possible candidates as CSF biomarkers.

## Author Contributions

VP planned and conducted all the experiments, data analysis and interpretation. NB contributed to performing some experiments and figure preparation. EP provided materials and contributed to data analysis. FA conceived, planned, interpreted and supervised the study and wrote the manuscript. All authors read and approved its final manuscript.

## Conflict of Interest Statement

The authors declare that the research was conducted in the absence of any commercial or financial relationships that could be construed as a potential conflict of interest.
